# Network analysis of comorbid internet addiction, anxiety, and depression symptoms among Chinese junior high school students

**DOI:** 10.3389/fpubh.2026.1816697

**Published:** 2026-07-08

**Authors:** Ting Yu, Hong-Lei Wang, Xiao-Peng Deng, Juan Sheng, Bi-Ting Liu, Xiang-Zhan Chen, Xian-Ming Yuan, Yi-Xin Jin, Yuan Wang, Xin-Yi Pang, Xin-Feng Zhang, Song Qin, Zhe-Ming Tu

**Affiliations:** 1 Mental Health Center of Yangtze University, Jingzhou, Hubei, China; 2 Mental Health Institute of Yangtze University, Jingzhou, Hubei, China; 3 Jingzhou Rongjun Special Care Hospital, Jingzhou, Hubei, China; 4 Jingzhou Mental Health Center, Jingzhou, Hubei, China

**Keywords:** anxiety, depression, internet addiction, network analysis, youth students

## Abstract

**Background:**

Internet addiction (IA) frequently co-occurs with anxiety and depression during adolescence. However, traditional latent variable models based on total scores often mask specific symptom-level interactions. This study utilized network analysis to elucidate the fine-grained associations between IA, anxiety, and depression symptoms among Chinese adolescents and to explore potential gender differences in these network structures.

**Methods:**

A cross-sectional study was conducted among 10,491 junior high school students (mean age = 13.88 years; 51.98% male) in Jingzhou City, China. Participants completed the Internet Addiction Test (IAT), the Generalized Anxiety Disorder-7 (GAD-7), and the Patient Health Questionnaire-9 (PHQ-9). A Gaussian Graphical Model (GGM) was estimated to visualize the symptom network. Centrality indices and bridge strength were calculated to identify key symptoms. The Network Comparison Test (NCT) was employed to assess gender differences in global strength and network structure.

**Results:**

The network analysis revealed a stable structure driven primarily by symptoms reflecting escapism and social withdrawal. Specifically, “Preferring online interaction to real-world intimacy” (IAT19) and “Using the internet to block disturbing thoughts” (IAT10) exhibited the highest bridge strength, directly connecting to core depressive symptoms such as anhedonia (PHQ1) and depressed mood (PHQ2). The NCT indicated that while the global strength of the network was invariant across genders (*p* > 0.05), the network structure differed significantly between males and females (*p* = 0.01), suggesting distinct pathways of symptom interaction.

**Conclusion:**

Escapism and the displacement of offline social interactions are critical mechanisms maintaining the comorbidity of IA and emotional distress. Interventions should prioritize targeting these bridge symptoms—specifically by improving emotion regulation strategies and enhancing real-world social connections—to disrupt the feedback loop between addiction and internalizing problems. The structural differences observed suggest that gender-sensitive approaches may be required for effective intervention.

## 1 Introduction

Adolescence is a critical developmental period characterized by increased vulnerability to mental health challenges and profound changes in social behaviors (1). With the pervasive integration of digital technology into daily life, problematic internet use (PIU), often referred to as Internet Addiction (IA), has emerged as a significant public health concern among adolescents (2). Extensive epidemiological studies have demonstrated that IA is rarely an isolated phenomenon; rather, it frequently co-occurs with internalizing disorders, most notably anxiety and depression (3, 4). A recent large-scale study on Chinese adolescents reported that those with IA exhibited significantly higher risks of anxiety and depression compared to their non-addicted peers (5).

The co-occurrence of IA with anxiety and depression is increasingly understood as the product of reciprocal, mutually reinforcing processes rather than incidental overlap. On one hand, adolescents experiencing anxious or depressive affect may turn to the online world for mood regulation, distraction, or social compensation, in line with mood-enhancement and compensatory internet-use perspectives (6). On the other hand, excessive internet use can displace sleep, physical activity, academic engagement, and face-to-face relationships, thereby intensifying loneliness and negative affect (7, 8). Anxiety and depression are themselves tightly interwoven during adolescence, sharing vulnerabilities such as emotion-regulation deficits, rumination, and heightened stress reactivity (9). Consistent with this, meta-analytic and large-scale epidemiological evidence indicates that adolescents with IA report substantially elevated anxiety and depressive symptoms (4, 5), and that these internalizing problems in turn predict the onset and persistence of problematic internet use (10). Such bidirectional, dynamic patterning is difficult to capture with aggregate sum-score models, providing a clear rationale for examining how individual symptoms of IA, anxiety, and depression are interconnected.

However, the majority of prior studies have relied on traditional latent variable models (e.g., correlation or regression analyses based on sum-scores) (11). These approaches assume that symptoms (e.g., “insomnia,” “nervousness,” “uncontrolled internet use”) are passive indicators of underlying latent constructs (12). While valuable for establishing overall comorbidity rates, these aggregate-level analyses mask the complex, granular interactions between specific symptoms (13). For instance, a total-score analysis might indicate that “depression is correlated with internet addiction,” but it cannot clarify whether “insomnia” directly triggers “excessive late-night internet use,” or if “social withdrawal” leads to “online escapism.” Understanding these fine-grained pathways is crucial for developing targeted interventions.

To address these limitations, the network perspective of psychopathology offers a powerful alternative framework (14, 15). In this view, mental disorders are conceptualized not as latent entities but as complex systems of mutually reinforcing symptoms (16). Within such a network, symptoms are “nodes,” and their associations are “edges.” This approach allows researchers to identify central nodes (symptoms that are most strongly connected to others and likely drive the disorder) and bridge nodes (symptoms that connect two different disorders, such as IA and depression) (17). Identifying these key nodes provides specific targets for clinical intervention, as deactivating a central or bridge node may collapse the entire symptom network and prevent comorbidity.

Despite the growing popularity of network analysis, few studies have simultaneously examined the symptom-level interplay of internet addiction, anxiety, and depression in a large-scale adolescent sample. Furthermore, while gender differences in the prevalence of IA and mood disorders are well-documented (18), it remains unclear whether the structure of these symptom interactions differs between male and female adolescents. Recent findings suggest that the pathways linking anxiety to internet use might differ by gender, indicating a need for gender-sensitive intervention strategies (19, 20).

The present study aims to fill these gaps by applying network analysis to a large sample of Chinese junior high school students (*N* = 10,491). Specifically, we aim to: (1) estimate the network structure of IA, anxiety, and depression to visualize detailed symptom-level associations; (2) identify the most central symptoms within the network and the bridge symptoms that connect IA with mental health problems; and (3) explore whether the global strength and network structure differ significantly between male and female students using the Network Comparison Test (NCT). By moving beyond aggregate scores, this study seeks to provide novel insights into the specific mechanisms maintaining the comorbidity of internet addiction and emotional distress in adolescents.

## 2 Methods

### 2.1 Study design and participants

A school-based cross-sectional study was conducted from March 2024 to April 2024. To avoid the methodological limitations associated with online convenience sampling, this study was coordinated in official partnership with the local Education Bureau, targeting all junior high schools within the Jingzhou District of Jingzhou City, China. Data collection utilized a supervised, school-based cluster sampling approach. A total of 20 junior high schools participated in the study. Rather than allowing students to complete the survey independently on personal devices, the digital questionnaires were administered uniformly in the schools’ computer laboratories. To ensure data integrity, authenticity, and focus, trained teachers and research assistants were physically present to supervise the students in real-time as they completed the assessments. A total of 12,254 questionnaires were initially collected under these controlled conditions. Following rigorous data quality control procedures, 10,491 valid questionnaires were retained, yielding an effective response rate of 85.61%. The exclusion criteria were strictly defined as follows: (1) questionnaires with a response duration of less than 232 s or more than 3,000 s; (2) responses with incorrect answers to embedded attention-check items (trap questions); and (3) duplicate submissions. The study protocol was approved by the Research Ethics Committee of the Jingzhou Mental Health Center (Approval No.: 2021LL0501). As all participants were minors, parents or legal guardians were notified of the study in advance through the schools and the local Education Bureau, and participation was entirely voluntary. Informed consent was obtained electronically from each student via the first item of the questionnaire, which asked whether they agreed to participate; students who selected “no” exited immediately and no data were collected from them. The Research Ethics Committee approved this consent procedure. The sample size was not derived from an *a priori* power analysis for a specific effect size; instead, it was determined by a whole-population (census-type) cluster-sampling frame. In cooperation with the local Education Bureau, all 20 junior high schools and all enrolled students in the Jingzhou District were invited to participate, and the analytic sample comprised every student who provided valid data. This approach yielded a sample that far exceeds the requirements for stable estimation of regularized partial-correlation networks (21), as confirmed by the case-dropping bootstrap (CS-coefficient for node and bridge strength = 0.75).

### 2.2 Internet addiction test (IAT)

Internet addiction was assessed using the Internet Addiction Test (IAT) developed by Young (22). This scale consists of 20 items that measure the severity of compulsive internet use and its impact on daily life (e.g., “How often do you find that you stay on-line longer than you intended?”). Participants rated each item on a 5-point Likert scale ranging from 1 (“Rarely”) to 5 (“Always”). The total score ranges from 20 to 100, with higher scores indicating a higher level of internet addiction. In the present study, the Cronbach’s alpha coefficient for the IAT was 0.934. The IAT has been validated in Chinese adolescent samples, showing good internal consistency and a stable factor structure in this age group, which supports its applicability to junior high school students (23).

### 2.3 Generalized anxiety disorder-7 (GAD-7)

Anxiety symptoms were measured using the Generalized Anxiety Disorder-7 (GAD-7) scale (24). This instrument consists of 7 items screening for generalized anxiety symptoms over the past 2 weeks (e.g., “Feeling nervous, anxious or on edge”). Items are rated on a 4-point Likert scale from 0 (“Not at all”) to 3 (“Nearly every day”). Total scores range from 0 to 21, with higher scores reflecting greater anxiety severity. In this study, the Cronbach’s alpha coefficient was 0.936. The GAD-7 has demonstrated sound psychometric properties and measurement invariance in large samples of Chinese adolescents aged 10–17 years, supporting its use in the present population (25).

### 2.4 Patient health questionnaire-9 (PHQ-9)

Depressive symptoms were assessed using the Patient Health Questionnaire-9 (PHQ-9) (26). The scale comprises 9 items corresponding to the DSM-IV diagnostic criteria for major depressive disorder. Participants indicated how often they had been bothered by each symptom over the last 2 weeks (e.g., “Little interest or pleasure in doing things”) on a 4-point scale ranging from 0 (“Not at all”) to 3 (“Nearly every day”). The total score ranges from 0 to 27, with higher scores indicating more severe depression. In the current study, the Cronbach’s alpha coefficient was 0.918. The PHQ-9 has likewise been widely used and validated as a screening measure of depressive symptoms among adolescents (27).

### 2.5 Data analysis

Descriptive statistics were computed using R version 4.3.1. Network analysis was conducted using the R packages qgraph (version 1.9.5), bootnet (version 1.5), networktools, and NetworkComparisonTest (NCT).

The network structure was estimated using the Gaussian Graphical Model (GGM). In this network, nodes represent the items of the IAT, GAD-7, and PHQ-9, while edges represent the partial correlations between two nodes after controlling for all other nodes in the network. To minimize spurious edges and ensure a sparse, interpretable network, we employed the Extended Bayesian Information Criterion (EBIC) with the Graphical Least Absolute Shrinkage and Selection Operator (LASSO) regularization (EBICglasso) (21). The tuning parameter *γ* was set to 0.5 to balance sensitivity and specificity. The visualization was generated using the Fruchterman-Reingold algorithm, which places strongly connected nodes closer together.

To identify the most influential symptoms, we calculated node Strength, which is the sum of the absolute weights of all edges connected to a node. While Closeness and Betweenness were also computed, we focused our interpretation primarily on Strength, following recent methodological recommendations that highlight the instability of Closeness and Betweenness in psychological networks (28).

The stability of the network was examined using the bootnet package. We performed two types of bootstrapping:

Non-parametric bootstrap (1,000 samples) was used to estimate the 95% confidence intervals (CIs) of the edge weights to assess their accuracy.

Case-dropping bootstrap was used to assess the stability of centrality indices. This is quantified by the correlation stability coefficient (CS-coefficient), which indicates the maximum proportion of cases that can be dropped while maintaining a correlation of at least 0.7 with the original indices in 95% of the samples. A CS-coefficient above 0.5 is considered ideal.

To explore gender differences, we used the Network Comparison Test (NCT) with 1,000 permutations (17). We tested for invariance in (1) Global Strength (the sum of all absolute edge weights in the network) and (2) Network Structure (the distribution of edge weights). If significant structural differences were found, we further examined specific edges using Holm-Bonferroni correction to control for multiple comparisons.

## 3 Results

### 3.1 Participants and descriptive statistics

The sample comprised 5,453 males (52.0%) and 5,038 females (48.0%). The mean age of participants was 13.88 years (SD = 0.92, range = 11–16 years). The participants were distributed across three grade levels: 4,050 students (38.6%) were in the first year (Grade 7), 3,516 students (33.5%) in the second year (Grade 8), and 2,925 students (27.9%) in the third year (Grade 9) of junior high school (Tables 1, 2).

**Table 1:** Demographic characteristics of the study participants (*N* = 10,491).

Characteristic	*N* = 10,491^1^
Age	13.88 (0.92)
Sex	
Females	5,038 (48.02%)
Males	5,453 (51.98%)
Ethnicity	
Han	10,142 (96.67%)
Other	349 (3.33%)
Grade	
Grade 7	4,050 (38.60%)
Grade 8	3,516 (33.51%)
Grade 9	2,925 (27.88%)

1 Mean (SD); *n* (%).

**Table 2:** Descriptive statistics for IAT, GAD-7, and PHQ-9.

Characteristic	Item wording	*N* = 10,491^1^
IAT1	Stay online longer than intended	2.00 (IQR: 1.00–3.00)
IAT2	Neglect household chores to spend more time online	2.00 (IQR: 1.00–2.00)
IAT3	Prefer the excitement of the Internet to intimacy with others	1.00 (IQR: 1.00–2.00)
IAT4	Form new relationships with fellow online users	1.00 (IQR: 1.00–2.00)
IAT5	Others complain about the amount of time spent online	2.00 (IQR: 1.00–3.00)
IAT6	Grades or school work suffer because of online time	1.00 (IQR: 1.00–2.00)
IAT7	Check email or messages before doing necessary tasks	1.00 (IQR: 1.00–2.00)
IAT8	Job performance or productivity suffer because of the Internet	1.00 (IQR: 1.00–2.00)
IAT9	Become defensive or secretive when asked what you do online	2.00 (IQR: 1.00–3.00)
IAT10	Block out disturbing thoughts with soothing thoughts of the Internet	2.00 (IQR: 1.00–3.00)
IAT11	Anticipate when you will go online again	2.00 (IQR: 1.00–2.00)
IAT12	Fear that life without the Internet would be boring and empty	1.00 (IQR: 1.00–2.00)
IAT13	Snap, yell, or act annoyed if bothered while online	1.00 (IQR: 1.00–2.00)
IAT14	Lose sleep due to late-night logins	1.00 (IQR: 1.00–2.00)
IAT15	Feel preoccupied with the Internet when offline	1.00 (IQR: 1.00–2.00)
IAT16	Say “just a few more minutes” when online	1.00 (IQR: 1.00–2.00)
IAT17	Try to cut down the amount of time spent online and fail	1.00 (IQR: 1.00–2.00)
IAT18	Try to hide how long you have been online	1.00 (IQR: 1.00–2.00)
IAT19	Choose to spend more time online over going out with others	1.00 (IQR: 1.00–1.00)
IAT20	Feel depressed or moody when offline, which goes away when back online	1.00 (IQR: 1.00–1.00)
PHQ1	Little interest or pleasure in doing things	0.00 (IQR: 0.00–1.00)
PHQ2	Feeling down, depressed, or hopeless	0.00 (IQR: 0.00–1.00)
PHQ3	Trouble falling or staying asleep, or sleeping too much	0.00 (IQR: 0.00–1.00)
PHQ4	Feeling tired or having little energy	0.00 (IQR: 0.00–1.00)
PHQ5	Poor appetite or overeating	0.00 (IQR: 0.00–1.00)
PHQ6	Feeling bad about yourself, or that you are a failure	0.00 (IQR: 0.00–1.00)
PHQ7	Trouble concentrating on things, such as reading or watching TV	0.00 (IQR: 0.00–0.00)
PHQ8	Moving or speaking so slowly (or too fast) that others could have noticed	0.00 (IQR: 0.00–0.00)
PHQ9	Thoughts that you would be better off dead or of hurting yourself	0.00 (IQR: 0.00–0.00)
GAD1	Feeling nervous, anxious, or on edge	0.00 (IQR: 0.00–1.00)
GAD2	Not being able to stop or control worrying	0.00 (IQR: 0.00–1.00)
GAD3	Worrying too much about different things	0.00 (IQR: 0.00–1.00)
GAD4	Trouble relaxing	0.00 (IQR: 0.00–1.00)
GAD5	Being so restless that it is hard to sit still	0.00 (IQR: 0.00–0.00)
GAD6	Becoming easily annoyed or irritable	0.00 (IQR: 0.00–1.00)
GAD7	Feeling afraid, as if something awful might happen	0.00 (IQR: 0.00–1.00)

1 Median (IQR: Q1–Q3).

Descriptive statistics for the measures of internet addiction, anxiety, and depression are presented in Table 1. The Internet Addiction Test (IAT) scores showed that the median endorsement for most items ranged between 1.00 and 2.00, suggesting a generally low-to-moderate level of problematic internet use in this sample. Regarding mental health symptoms, the median scores for all items on the Generalized Anxiety Disorder Scale (GAD-7) and the Patient Health Questionnaire (PHQ-9) were 0.00 (IQR: 0.00–1.00), indicating a positively skewed distribution where the majority of students reported low frequency of anxiety and depression symptoms.

### 3.2 Network structure

**Figure 1:**
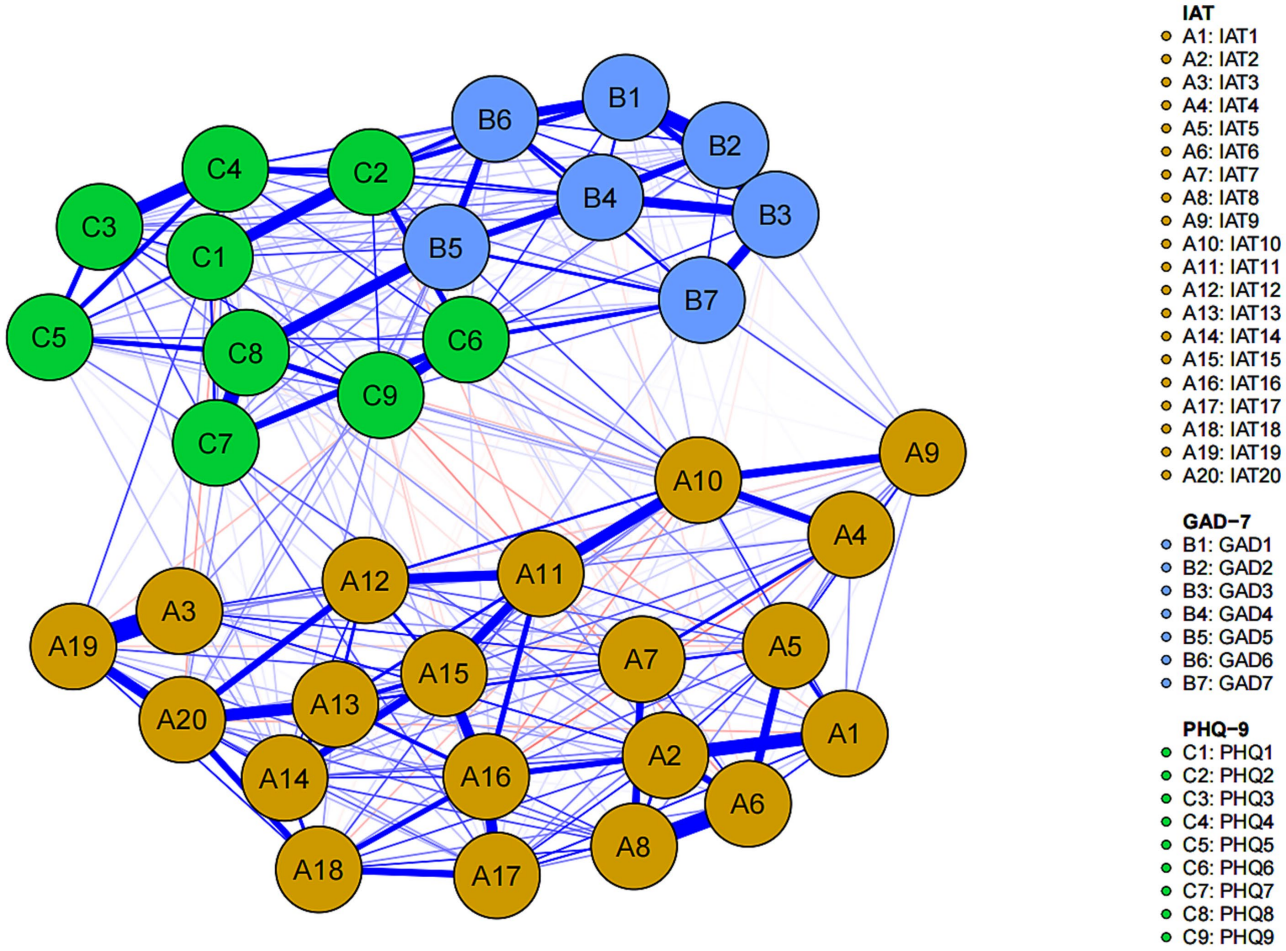
Association network of IAT, GAD-7 and PHQ-9.

**Figure 2:**
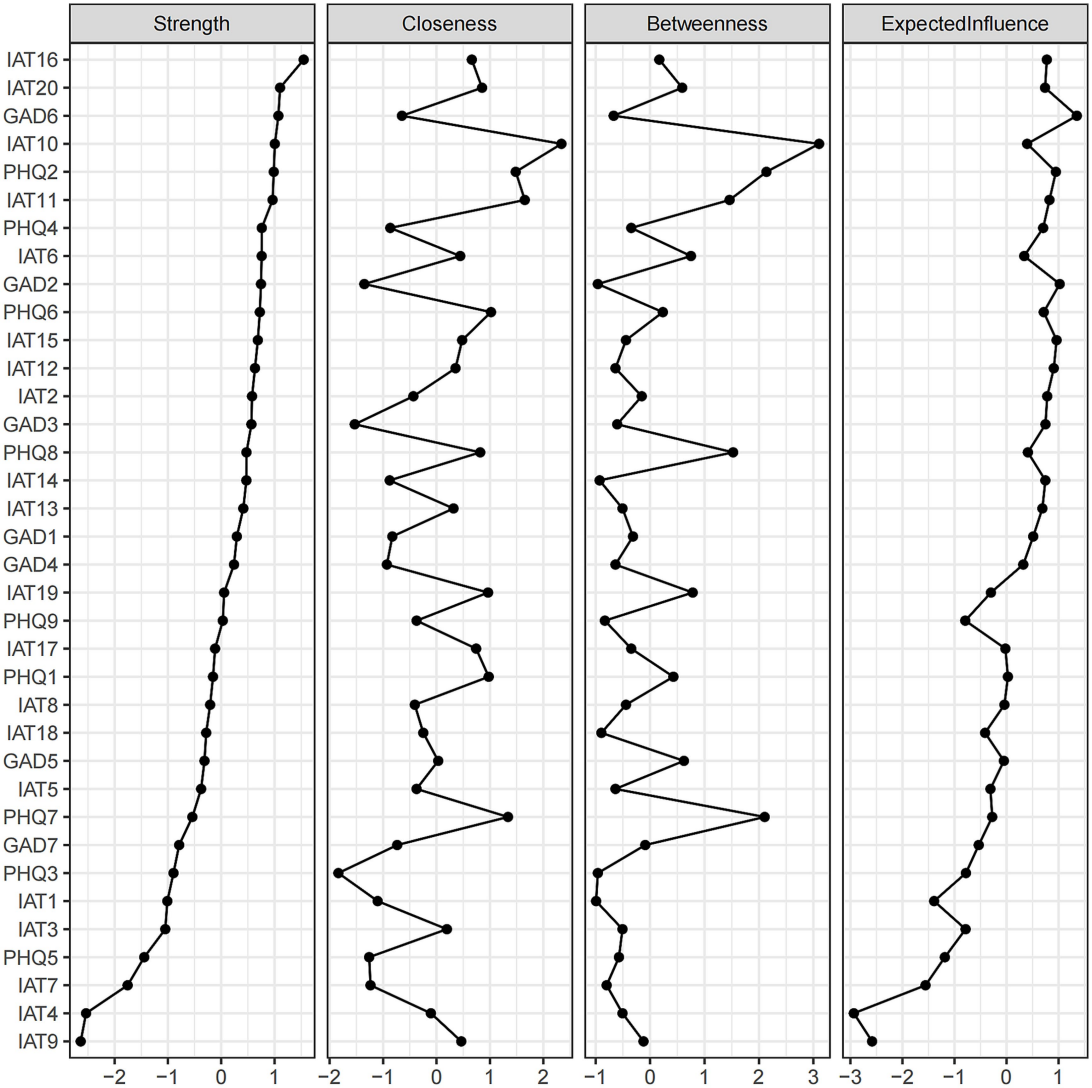
Bridge centrality indices shown as standardized *z*-scores.

The psychometric network structure of internet addiction, anxiety, and depression is presented in Figures 1, 2. The network was estimated using the EBICglasso algorithm, which effectively minimizes spurious correlations.

Within the IAT community, strong positive edges were observed between items reflecting similar domains of problematic use. The strongest edge in the entire IAT network was between item IAT3 (“Prefer online to intimacy”) and item IAT19 (“Prefer online to going out with friends”) (weight = 0.36), indicating a strong clustering of symptoms related to social withdrawal and preference for online interaction. Another prominent connection was found between IAT6 (“School grades suffering”) and IAT8 (“Job/daily life suffering”) (weight = 0.30), reflecting the functional impairment associated with internet use.

Within the mental health communities (GAD-7 and PHQ-9), strong connections were observed both within and between the two scales. For instance, strong edges linked anxiety symptoms such as GAD1 (“Nervousness”) and GAD2 (“Uncontrollable worry”) (weight = 0.27). Notably, there were substantial edges connecting the anxiety and depression communities, such as the link between GAD1 (“Nervousness”) and PHQ2 (“Feeling down/depressed”) (weight = 0.13), which was stronger than any edge connecting these symptoms to the IAT network.

Regarding the edges bridging internet addiction and mental health symptoms, the connections were generally positive but weaker than the within-community edges. The strongest bridge edges primarily involved IAT items related to escapism and social withdrawal. Specifically, IAT19 (“Prefer online to friends”) showed a direct positive link with PHQ1 (“Little interest or pleasure”) (weight = 0.06). Additionally, IAT10 (“Block disturbing thoughts with internet”) was connected to PHQ2 (“Feeling down/depressed”) (weight = 0.05) and PHQ6 (“Feeling bad about yourself”) (weight = 0.05). These edges suggest that participants who use the internet to escape from negative feelings or who prioritize online interaction over social life are more likely to report concurrent core symptoms of depression.

### 3.3 Network stability

The stability of the network structure and centrality indices was assessed using the case-dropping bootstrap procedure. The stability of centrality indices is quantified by the correlation stability coefficient (CS-coefficient). Ideally, the CS-coefficient should be above 0.5.

**Figure 3:**
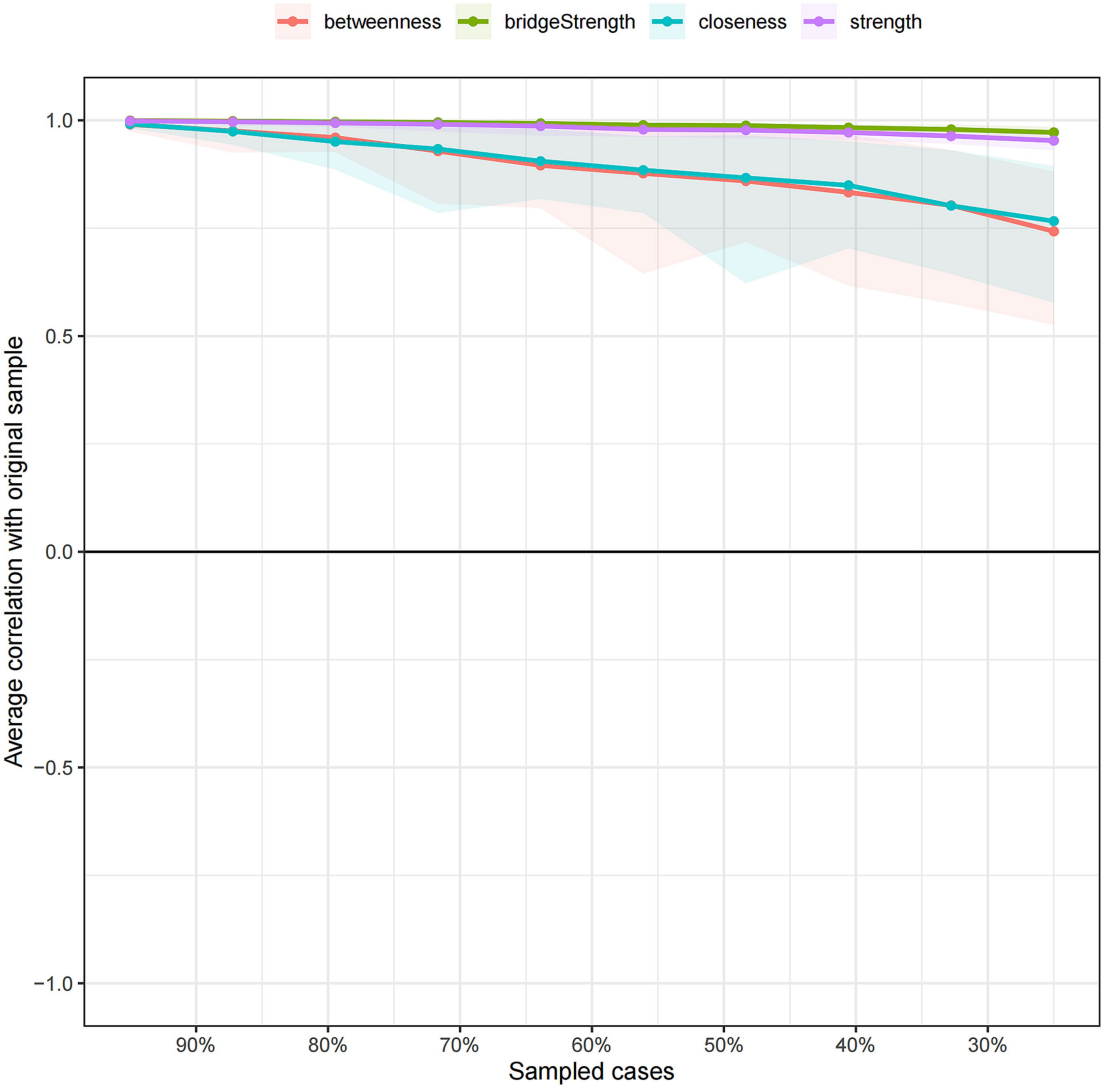
Stability of centrality indices by case dropping subset bootstrap.

As shown in Figure 3, the stability analysis indicated that the estimated network was highly stable. The CS-coefficients for node strength, closeness, and betweenness were 0.75, 0.594, and 0.517, respectively. Although all indices exceeded the threshold, we primarily focus our interpretation on node Strength (CS = 0.75) and Bridge Strength (CS = 0.75) in the subsequent discussion. This decision aligns with recent methodological recommendations arguing that Closeness and Betweenness are less reliable in psychological networks (28, 29).

In addition, non-parametric bootstrapping (1,000 samples) was performed to assess the accuracy of edge weights. The results indicated that the edge weight estimates were sufficiently accurate, with narrow confidence intervals around the estimated edge weights (see Supplementary Material).

### 3.4 Network comparisons

The Network Comparison Test (NCT) was conducted to examine potential differences in network structure and global strength between male and female students.

First, the Global Strength Invariance Test revealed no significant difference in the overall level of connectivity between the two groups (S = 0.32, *p* = 0.28). This suggests that the total sum of edge weights, reflecting the overall cohesion of the symptom network, remains stable across genders.

However, the Network Structure Invariance Test indicated a statistically significant difference in the network organization (M = 0.13, *p* = 0.01). This finding implies that while the overall connectivity is similar, the specific configuration of symptom interactions—how certain symptoms relate to others—differs between male and female students.

Despite the global structural difference, subsequent tests for specific edge weights and centrality indices (strength) did not yield any statistically significant results after correcting for multiple comparisons (Holm-Bonferroni correction, all *p* > 0.05). This pattern suggests that the observed structural difference is likely driven by a combination of subtle variations across multiple pathways in the network rather than by a substantial disparity in a single specific edge or symptom. This indicates that while the overall symptom burden is comparable, the specific pathways maintaining these associations may vary slightly by gender.

## 4 Discussion

The present study applied network analysis to a large-scale sample of Chinese junior high school students (*N* = 10,491) to elucidate the complex symptom-level interplay between internet addiction (IA), anxiety, and depression. By moving beyond traditional latent variable models, we identified specific pathways connecting problematic internet use with internalizing symptoms and explored gender differences in these network structures. The results revealed a highly stable network where symptoms of IA and depression are bridged primarily through mechanisms of escapism and social withdrawal.

### 4.1 The role of bridge symptoms: escapism and social withdrawal

One of the most significant findings of this study is the identification of key bridge nodes connecting the IA community to the depression community. Specifically, IAT19 (“Prefer online to going out with friends”) and IAT10 (“Block disturbing thoughts with internet”) exhibited the highest bridge strength.

The strong link between IAT10 (Escapism) and depressive symptoms (e.g., PHQ2 “Feeling down/depressed”) aligns with the Compensatory Internet Use Theory (CIUT) (6) and the Self-Medication Hypothesis (30, 31). These theories posit that individuals do not become addicted to the internet per se, but rather utilize online activities as a maladaptive coping strategy to alleviate negative affect or escape from real-life difficulties. In our network, this pathway suggests a potential vicious cycle: adolescents experiencing depressive symptoms (e.g., low self-worth, sadness) may turn to the internet to “block” these disturbing thoughts (IAT10). While this provides temporary relief, it reinforces the reliance on the internet, thereby maintaining the psychopathological network. Recent network studies have similarly identified escapism as a transdiagnostic mechanism linking addictive behaviors with mood disorders (32).

Junior high school is a critical period for social development and peer bonding (33). Students who find online interactions more rewarding than offline intimacy (IAT19) may gradually withdraw from real-world social support systems. This isolation is closely linked to anhedonia and loneliness, which are core components of depression (34, 35). Our findings corroborate previous longitudinal research suggesting that the displacement of real-world social interaction by virtual interaction is a key mechanism driving the comorbidity of IA and depression (7, 8).

These findings both converge with and extend the small but growing body of network studies on internet use and emotional distress. A recent network analysis of internet addiction and depression in Chinese adolescents similarly identified escapism-related items (32) as pivotal in linking the two domains, mirroring the prominence of IAT10 in our network. Work mapping internet addiction onto residual depressive symptoms in clinical samples has likewise highlighted interest- and mood-related symptoms such as anhedonia as key points of contact (20), consistent with the bridges we observed between IAT19/IAT10 and PHQ1/PHQ2. At the same time, our results refine this picture. First, in this non-clinical, school-based sample the cross-community bridges were positive but modest relative to within-scale edges, suggesting that in the general adolescent population IA and internalizing symptoms remain partially distinct systems coupled through a limited set of escapism and social-withdrawal pathways rather than being densely fused. Second, that the anxiety–depression edges (e.g., GAD1–PHQ2) were stronger than any IA–mood edge indicates that anxiety and depression constitute the affective core of the network, with problematic internet use attaching to this core mainly through maladaptive coping. Together, these comparisons position escapism and offline social displacement as candidate transdiagnostic mechanisms that appear reproducible across samples, while their magnitude may scale with clinical severity.

### 4.2 Network structure and gender differences

Our results from the Network Comparison Test (NCT) offer a nuanced view of gender differences. We found no significant difference in Global Strength, indicating that the overall severity and cohesion of the symptom network are comparable between male and female students. This finding contradicts some prior studies that reported higher network connectivity in females (36), but aligns with recent large-scale evidence suggesting that the “symptom burden” is becoming increasingly similar across genders in the digital age (37).

However, the significant difference in Network Structure (*p* = 0.01) implies that the specific configuration of edges—the “wiring” of the network—differs by gender. Although specific edge differences did not survive multiple testing corrections, the structural divergence likely reflects distinct motivations for internet use. Literature consistently indicates that males are more likely to engage in online gaming for achievement and dominance, whereas females are more inclined to use social media for relational maintenance and social comparison (10, 38, 39). Consequently, the pathways linking anxiety (e.g., social evaluative threat) to internet use may differ: females might be driven by “Fear of Missing Out” (FoMO) (40), while males might be driven by the need for escapism through immersive gaming (41). This structural heterogeneity suggests that while the outcome (comorbidity) is the same, the underlying psychological mechanisms may require gender-sensitive interpretations.

### 4.3 Clinical implications

From a clinical perspective, identifying bridge symptoms provides actionable targets for intervention. According to the Network Theory of Psychopathology, targeting bridge nodes is more effective than targeting peripheral symptoms because deactivating bridge nodes can prevent the spread of activation between disorders, potentially collapsing the comorbidity network (12, 42).

Our findings suggest that interventions should prioritize:

Addressing Maladaptive Coping (Targeting IAT10): Clinicians should focus on emotion-regulation strategies (e.g., mindfulness, cognitive restructuring) to help students manage negative affect without resorting to digital escapism (43).

Enhancing Real-world Connection (Targeting IAT19): Interventions aiming to improve offline social skills and peer support may buffer against the cycle of social withdrawal and anhedonia (44).

### 4.4 Limitations

Several limitations should be noted. First, the study utilized a cross-sectional design, which precludes causal inferences. While network analysis generates hypotheses about potential pathways (e.g., depression to escapism to IA), we cannot determine the directionality of edges without longitudinal data (45, 46). Future studies should employ Cross-Lagged Panel Network (CLPN) models to confirm these temporal dynamics.

Second, all data were collected via self-report measures, making the results susceptible to social desirability bias and shared method variance (47). Adolescents might underreport mental health symptoms due to perceived stigma.

Third, the sample was restricted to junior high school students in China. Cultural factors, such as academic pressure and parenting styles, may influence the network structure, limiting generalizability to Western populations (48).

### 4.5 Future directions

Building on these findings, several directions merit attention. First, longitudinal designs and Cross-Lagged Panel Network (CLPN) models (45, 46) are needed to test the directionality of the escapism–depression and social-withdrawal pathways suggested here and to clarify whether internalizing symptoms precede, follow, or co-evolve with problematic internet use. Second, experimental and intervention studies should directly test whether targeting the identified bridge symptoms (IAT10 and IAT19)—for example, through emotion-regulation training (43) and programs that rebuild offline social connection (44)—produces broader de-activation of the comorbidity network, thereby validating the clinical utility of the network approach. Third, future work should incorporate multi-informant and objective indicators (e.g., parent or teacher reports and device-based usage data) to reduce reliance on self-report and shared-method variance. Fourth, replication across developmental stages (e.g., primary-school children and senior high or university students), clinical versus community samples, and different cultural contexts would establish the generalizability and boundary conditions of the present network. Finally, integrating relevant moderators and contextual factors—such as academic pressure, sleep, family environment, and patterns of online activity (e.g., gaming versus social media)—may help explain the gender-related structural differences observed and inform more precise, person-tailored interventions.

## Data Availability

The raw data supporting the conclusions of this article will be made available by the authors, without undue reservation.
