# Supplementary figures and images for: Network analysis of comorbid internet addiction, anxiety, and depression symptoms among Chinese junior high school students
Source: Front Public Health. 2026 Jul 8;14:1816697. doi: 10.3389/fpubh.2026.1816697 (PMC13388152; doi:10.3389/fpubh.2026.1816697)

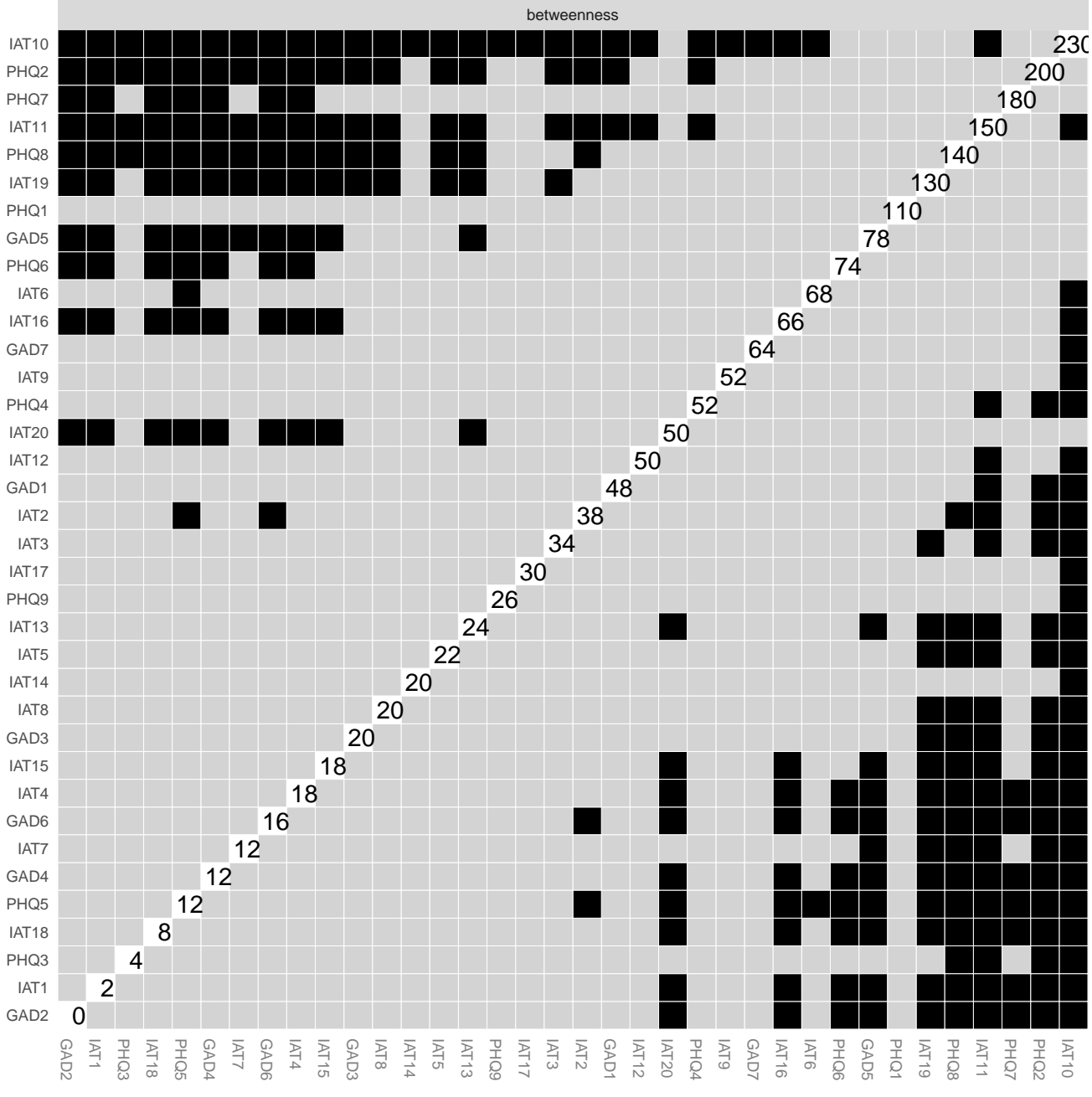

Supplement: Supplementary file 1 [file Data_Sheet_1.PDF]

Bridge Strength

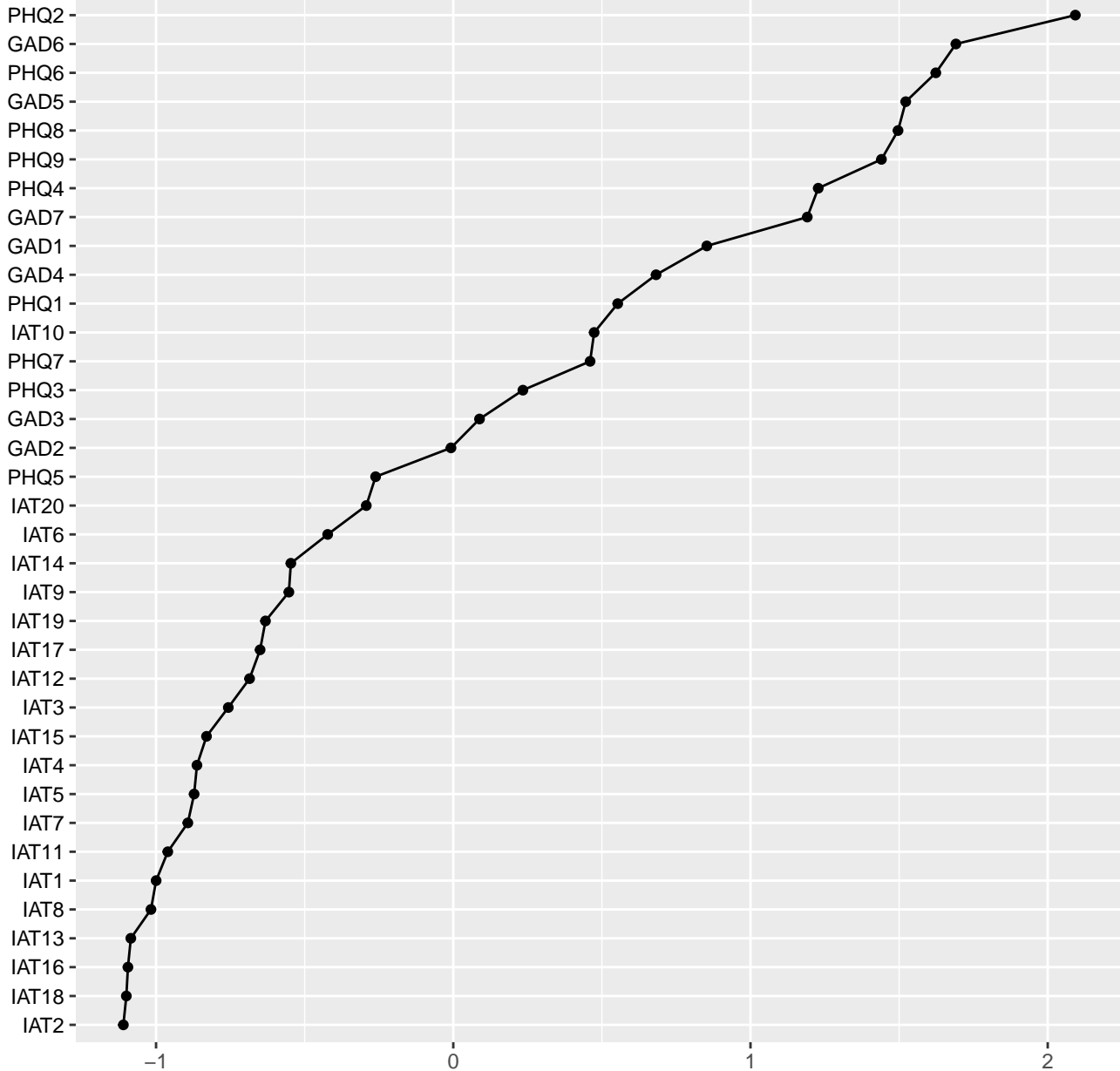

Supplement: Supplementary file 2 [file Data_Sheet_2.PDF]

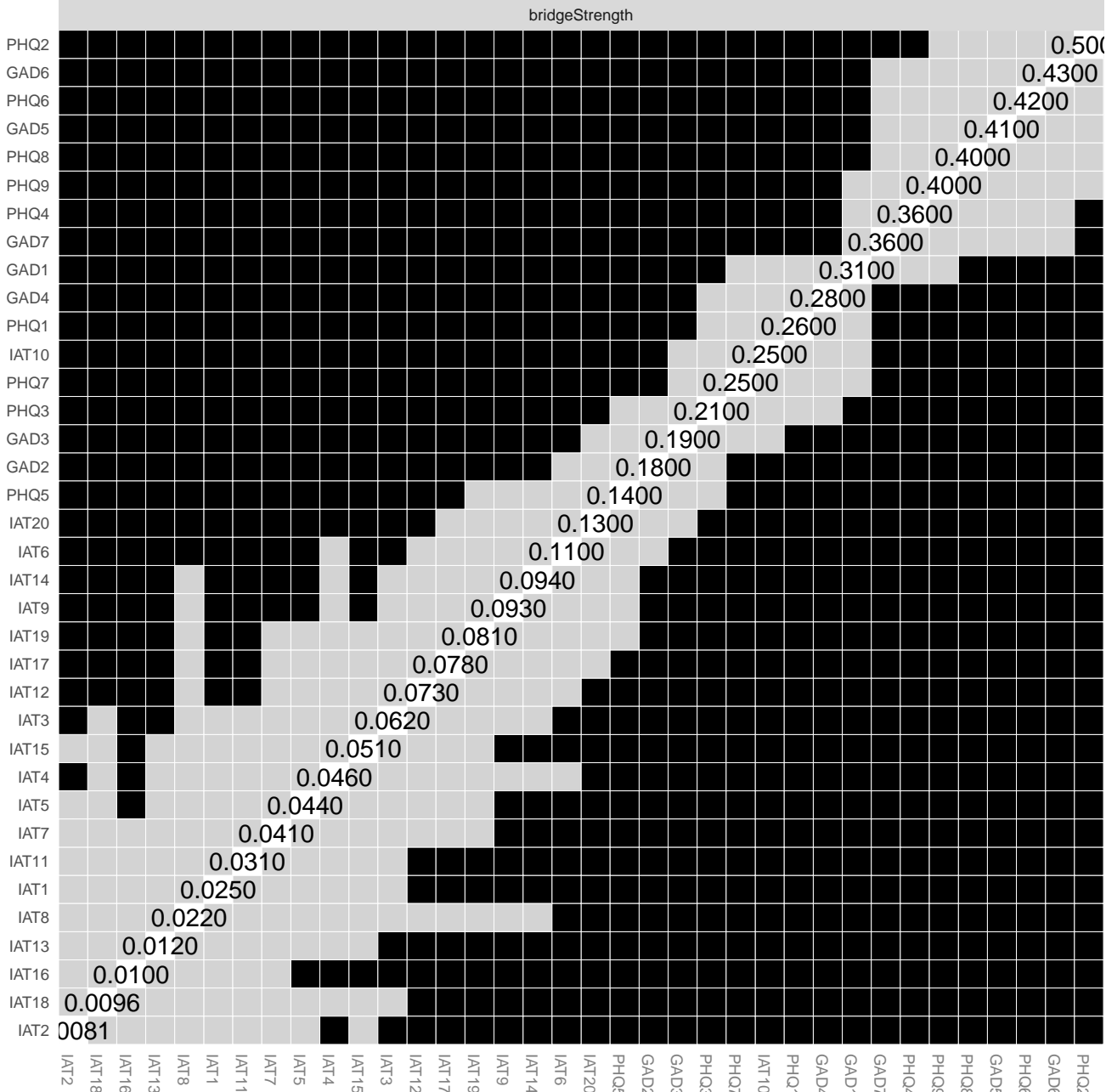

Supplement: Supplementary file 3 [file Data_Sheet_3.PDF]

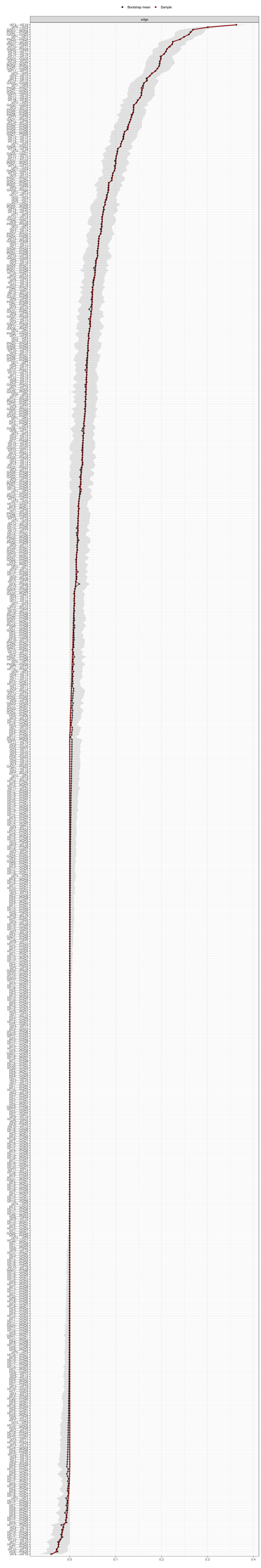

Supplement: Supplementary file 4 [file Data_Sheet_4.PDF]

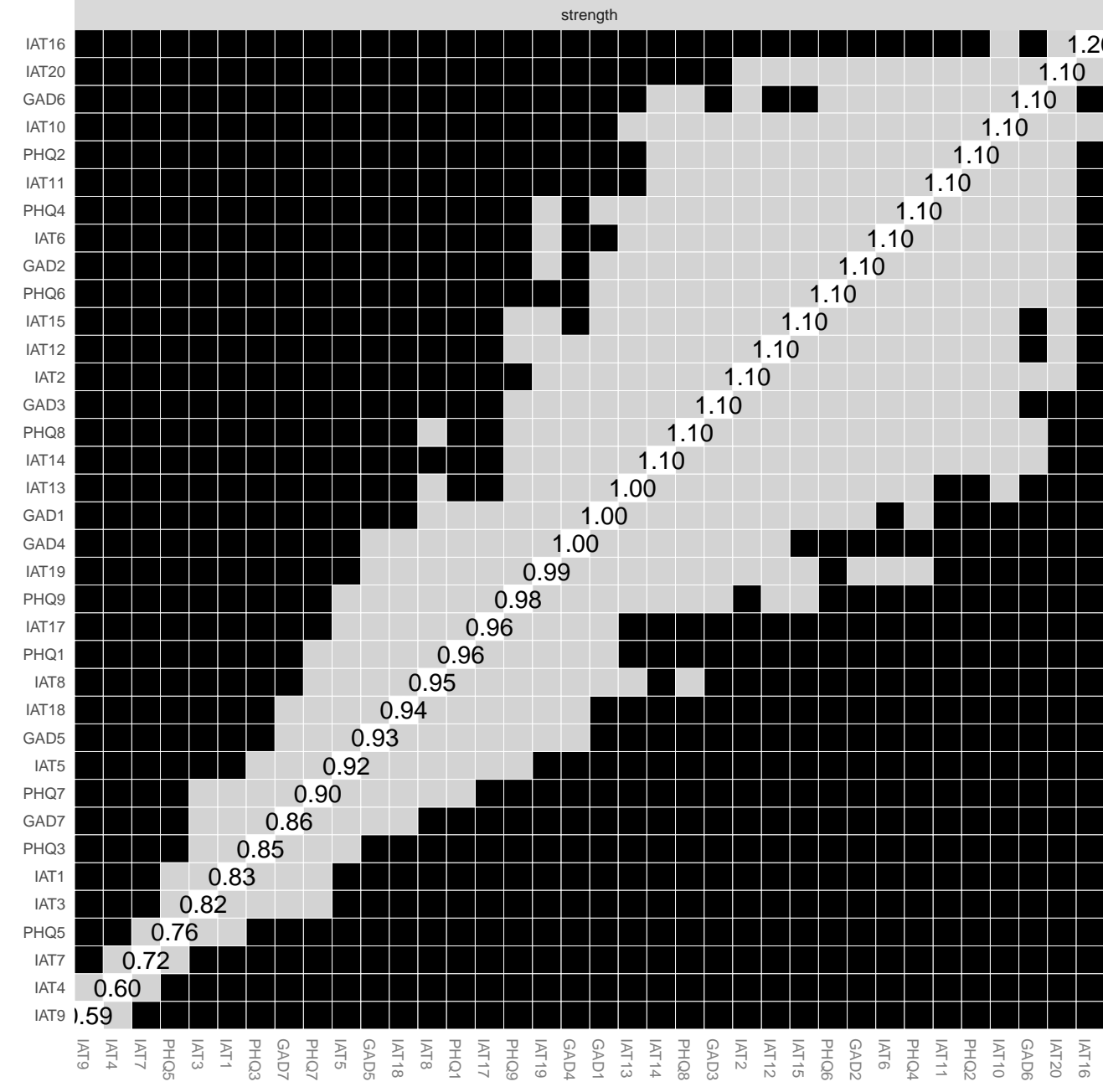

Supplement: Supplementary file 5 [file Data_Sheet_5.PDF]
